# Supplementary material for: Identifying genetic modifiers of age-associated penetrance in X-linked dystonia-parkinsonism
Source: Nat Commun. 2021 May 28;12:3216. doi: 10.1038/s41467-021-23491-4 (PMC8163740; doi:10.1038/s41467-021-23491-4)
Supplement: Supplementary file 1 — Supplementary Information [file 41467_2021_23491_MOESM1_ESM.pdf]

Supplementary Table 1. Individuals investigated in the present study

Total number of initially genotyped samples from male XDP patients	458
Excluded due to a low genotyping quality	8
Excluded due to relatedness to the included individuals	97
Patients included in the GWAS analysis	353
Previously reported patients out of those included in GWAS[1–4]	332/353
Patients in whom association of AAO and the hexanucleotide repeat number was previously reported[3,4]	328/353
Patients in whom *MSH3* exon 1 was sequenced out of those included in GWAS	285/353
Additional patients (not included in GWAS) in whom *MSH3* exon 1 was sequenced	95
Healthy Filipino controls without the SVA retrotransposon insertion	162

XDP - X-linked dystonia-parkinsonism; GWAS – genome-wide association study; AAO – age at onset; SVA - SINE-VNTR-Alu

Supplementary Table 2. Loci harboring single-nucleotide polymorphisms (SNPs) with genome-wide significance. Source data are provided as a Source Data file.

Chromosome	Region	Position	Number of SNPs	Number of exonic SNPs
5	Intergenic, 3' from *DHFR*	79893519 - 79920073	8	n/a
	DHFR	79928518 - 79947431	4	0
	MSH3	79950781 - 80171134	34	1
	Intergenic, 3' from *MSH3*	80181147 - 80222980	14	n/a
7	*EIF2AK1*	6064799 - 6098861	16	0
	ANKRD61, EIF2AK1	6071007 - 6076017	4	2 (*ANKRD61*)
	Intergenic, 5' from *EIF2AK1*	6098861 - 6106731	13	n/a

Supplementary Table 3. Variants identified in the X-linked dystonia-parkinsonism (XDP) patient cohort and their frequencies (in XDP patients, Filipino controls, and the Genome Aggregation Database (gnomAD) populations[5] (overall, East Asian, and South Asian) and impact on age at onset (AAO) adjusted for the hexanucleotide repeat number (using linear regression model; source data: provided as a Source Data file and https://gnomad.broadinstitute.org/gene/ENSG00000113318?dataset=gnomad_r2_1)

Chromosome	Position GRCh37	rsID	Reference	Alternate	Consequence - cDNA level	Consequence - protein level	Variant type
5	79950490	rs555608568	C	T	c.-57C>T	n.a.	5' UTR variant
5	79950497	rs2250063	C	T	c.-50C>T	n.a.	5' UTR variant
5	79950508	rs1105525	C	T	c.-39C>T	n.a.	5' UTR variant
5	79950512	rs1105524	A	G	c.-35A>G	n.a.	5' UTR variant
5	79950567	rs6151597	G	A	c.21G>A	p.Ala7Ala	synonymous
5	79950699	rs755000466	TGCAGCGGCTGCAGCGGCC	T	c.162_179delTGCAGCGGCCGCAGCGGC	p.Ala57_Ala62del	inframe deletion
5	79950708	rs2405875	T	C	c.162T>C	p.Ala54Ala	synonymous
5	79950724	rs2001675	G	GCCGCAGCGC	c.181_189dupGCAGCGCCC	p.Ala61_Pro63dup	inframe duplicat.
5	79950736	rs2405877	C	G	c.190C>G	p.Pro64Ala	missense
5	79950741	rs761734241	GCCCCCAGCT	G	c.199_207delCCAGCTCCC	p.Pro67_Pro69del	inframe deletion
5	79950781	rs1650697	A	G	c.235A>G	p.Ile79Val	missense
5	80047076	rs245013	A	C	n.a.	n.a.	intron 12 variant
5	80171134	rs33003	A	G	n.a.	n.a.	intron 23 variant
7	6070528	rs62456190	C	T	n.a.	n.a.	intron 11variant (*EIF2AK1*)

XDP patients		Filipino controls (no XDP haplotype)		gnomAD allele overall		gnomAD East Asian		gnomAD South Asian		AAO adjusted for the hexanucleotide repeat number	
Allele frequency	Homozygote count	Allele frequency	Homozygote count	Allele frequency	Homozygote count	Allele frequency	Homozygote count	Allele frequency	Homozygote count	Effect (β) ± standard error (in years)	p-value
0.0368	3/380	0.0340	0/162	0.0003	0	0.0043	0	0.0000	0	+0.29 ± 1.02	0.780612
0.0855	2/380	0.1142	6/162	0.2456	7002	0.0402	17	0.3380	1570	+3.06 ± 0.75	0.000048
0.0632	3/380	0.0586	1/162	0.1542	3233	0.0673	32	0.2386	838	-3.78 ± 0.81	0.000005
0.4908	88/380	0.4475	31/162	0.6465	49613	0.3612	924	0.7608	8016	-1.69 ± 0.42	0.000064
0.0053	0/380	0.0031	0/162	0.0076	10	0.0002	0	0.0164	6	n.a.	n.a.
0.1145	2/380	0.1759	6/162	0.2485	7702	0.0522	27	0.3404	1671	+2.15 ± 0.68	0.001673
0.0066	0/380	0.0031	1/162	0.0067	7	0.0011	0	0.0124	4	n.a.	n.a.
0.3434	38/380	0.2747	9/162	0.0531	367	0.0886	85	0.0440	40	-1.86 ± 0.45	0.000046
0.0039	0/380	0.0031	0/162	0.0169	9	0.0151	1	0.0174	0	n.a.	n.a.
0.1145	2/380	0.1759	6/162	0.2965	8405	0.0613	40	0.3911	1827	+2.15 ± 0.68	0.001673
0.5987	133/380	0.6111	54/162	0.8382	39707	0.7815	1669	0.8358	4972	+2.76 ± 0.41	0.000000
0.4412	124/376	0.3905	58/162	0.3550	2120	0.4141	138	0	0	-3.11 ± 0.44	0.000000
0.3964	165/376	0.4645	71/162	0.6094	5267	0.6316	295	0	0	-3.23 ± 0.44	0.000000
0.1652	251/376	0.1563	105/162	0.3576	2235	0.3784	116	0	0	3.86 ± 0.58	0.000000

n.a. - not applicable

Supplementary Table 4. *MSH3* exon 1 region haplotypes observed in X-linked dystonia-parkinsonism (XDP) patients and their association with ages at disease onset, using haplotype-based score statistics in 380 XDP patients. Source data are provided as a Source Data file.

Haplotype	rs555608568 c.-57C>T	rs2250063 c.-50C>T	rs1105525 c.-39C>T	rs1105524 c.-35A>G	Length polymorphism type	rs1650697 p.Ile79Val c.235A>G	Frequency	Hap-score	*p*-value	Effect (*β*) ± standard error (in years)	Haplotype counts
H1	C	C	C	A	6a	G	0.481	4.030	0.00006	+1.70 ± 0.41	362
H2	C	C	C	G	7a	A	0.290	-4.063	0.00005	-1.94 ± 0.47	224
H3	C	T	C	G	3a	G	0.083	4.174	0.00003	+3.21 ± 0.75	63
H4	C	C	T	G	6a	A	0.059	-4.281	0.00002	-3.70 ± 0.84	45
H5	T	C	C	G	7a	A	0.037	0.279	0.78020	+0.29 ± 1.02	28
H6	C	C	C	A	3a	G	0.024	-0.375	0.70799	-0.57 ± 1.53	22

Supplementary Table 5. Expression quantitative trait loci (eQTL) query of the lead single-nucleotide polymorphisms (SNPs) from our study using the BrainSeq dataset[6] from the eQTL Catalogue[7] Source data: https://www.ebi.ac.uk/eqtl/Studies/, https://www.gtexportal.org/home/gene/MSH3, https://www.gtexportal.org/home/gene/PMS2

Variant	Allele count	Alternative alleles	Effect (*β*) ± standard error	*p*-value	Effect direction in GTEx
rs245013	958	282	0.0145 ± 0.0145	0.3187	same
rs33003	958	411	0.0060 ± 0.0138	0.6636	not found in GTEx
rs1650697	958	148	-0.0331 ± 0.0184	0.0716	same
rs62456190	not assayed	not assayed	not assayed	not assayed	not assayed

GTEx - The Genotype-Tissue Expression (GTEx) Portal[8]

Supplementary Table 6. Expression analyses in blood of 40 X-linked dystonia-parkinsonism (XDP) patients using linear regression model. Source data are provided as a Source Data file and https://www.gtexportal.org/home/gene/MSH3, https://www.gtexportal.org/home/gene/PMS2.

A. Correlation of the lead SNPs on chromosome 5 and *MSH3* exon 1 polymorphisms with *MSH3* expression

Variant	Allele count	Alternative alleles	Effect (*β*) ± standard error	*p*-value	Effect direction in GTEx
rs245013	80	46	0.2084 ± 0.0966	0.0374	same
rs33003	80	58	-0.0212 ± 0.0860	0.8060	not found in GTEx
rs1650697	80	36	-0.2274 ± 0.0906	0.0164	same
3a	80	6	-0.0168 ± 0.1596	0.7373	not applicable
6a	80	32	-0.0920 ± 0.0715	0.1130	not applicable
7a	80	18	0.0512 ± 0.0774	0.0410	not applicable

SNP - single-nucleotide polymorphisms; GTEx - The Genotype-Tissue Expression Portal[8]

B. Correlation of the lead SNP on chromosome 7 with *PMS2* expression

Variant	Allele count	Alternative alleles	Effect (β) ± standard error	*p*-value	Effect direction in GTEx
rs62456190	80	12	0.01382 ± 0.1899	0.9420	same

SNP - single-nucleotide polymorphisms; GTEx - The Genotype-Tissue Expression Portal[8]

Supplementary Table 7. Primers and PCR conditions

<table>
<tr><th>PCR product</th><th>Primers</th><th>Reaction</th><th>Cycling conditions</th></tr>
<tr><td rowspan="2">Repeat analysis*</td><td>5’-[FAM]AGCAGTACAGTCCAGCTTTGGC-3’</td><td rowspan="2">2.5µl DNA 50ng/µl
5.5µl HPLC water
1.0µl PCR buffer
0.2µl 10xdNTPs
0.3µl Primer 1 (10µM)
0.3µl Primer 2 (10µM)
0.1µl PrimeSTAR GXL
(Takara Bio, Cat. #R050B)</td><td rowspan="2">94°C 2 min.;
30x (98°C 10 sec.,
64°C 35 sec.);
72°C 7 min.</td></tr>
<tr><td>5’-CTCAAGCCTTATTACAATGCCAGT-3’</td></tr>
<tr><td rowspan="2">MSH3 exon 1</td><td>5’-GGCAGGTCCCCGTTCTTGC-3’</td><td rowspan="2">1.0µl DNA 50ng/µl
4.5µl HPLC water
2.0µl GC-rich PCR reaction buffer, with 7.5mM $MgCl_2$ and DMSO 5x concentrated
0.2µl 10xdNTPs
0.4µl Primer 1 (10µM)
0.4µl Primer 2 (10µM)
0.1µl GC-rich Enzyme Mix
0.4µl $MgCl_2$ Stock solution 25mM
1.0µl GC-rich resolution solution 5M
(Roche GC-rich PCR System, Cat. #12140306001)</td><td rowspan="2">95°C 3 min.;
10x (95°C 30 sec.,
63°C 30 sec.,
72°C 30 sec.), 25x
(95°C 30 sec.,
63°C 30 sec.,
72°C 45 sec.);
72°C 7 min.</td></tr>
<tr><td>5’-CCCCTTCCTCCTCCAGCCC-3’</td></tr>
<tr><td>MSH3 cDNA ex12 F</td><td>5’-GCTGTGGGTTTTAGACCAC-3’</td><td rowspan="8">1.0µl cDNA
3.4µl HPLC water
0.3µl Primer 1 (10µM)
0.3µl Primer 2 (10µM)
5.0µl SYBR Green Mix
(thermoscientific, Cat. #K0241, Maxima SYBR Green/ Fluorescein qPCR Master Mix)</td><td rowspan="8">95°C 3 min.;
45x (95°C 15 sec.,
60°C 15 sec.,
72°C 30 sec.)</td></tr>
<tr><td>MSH3 cDNA ex14 R</td><td>5’-GGTATTATTGCTTGAAATTCTGAC-3’</td></tr>
<tr><td>PMS2-LC-ex6F</td><td>5’-TGGAAGCCCCAGCATAAAGG-3’</td></tr>
<tr><td>PMS2-LC-ex7R</td><td>5’-ATCGGAACAGCTCAAACCGT-3’</td></tr>
<tr><td>GAPDH_fwd_Ex1</td><td>5’-GTCAGCCGCATCTTCTTTTG-3’</td></tr>
<tr><td>GAPDH_rev_Ex3</td><td>5’-GCGCCCAATACGACCAAATC-3’</td></tr>
<tr><td>YWHAZ_LC_F</td><td>5’-GTAGGTCATCTTGGAGGGTCG-3’</td></tr>
<tr><td>YWHAZ_LC_R</td><td>5’-GCTATGCTTGTTGTGACTGATCG-3’</td></tr>
</table>

*Primers and conditions from [3].

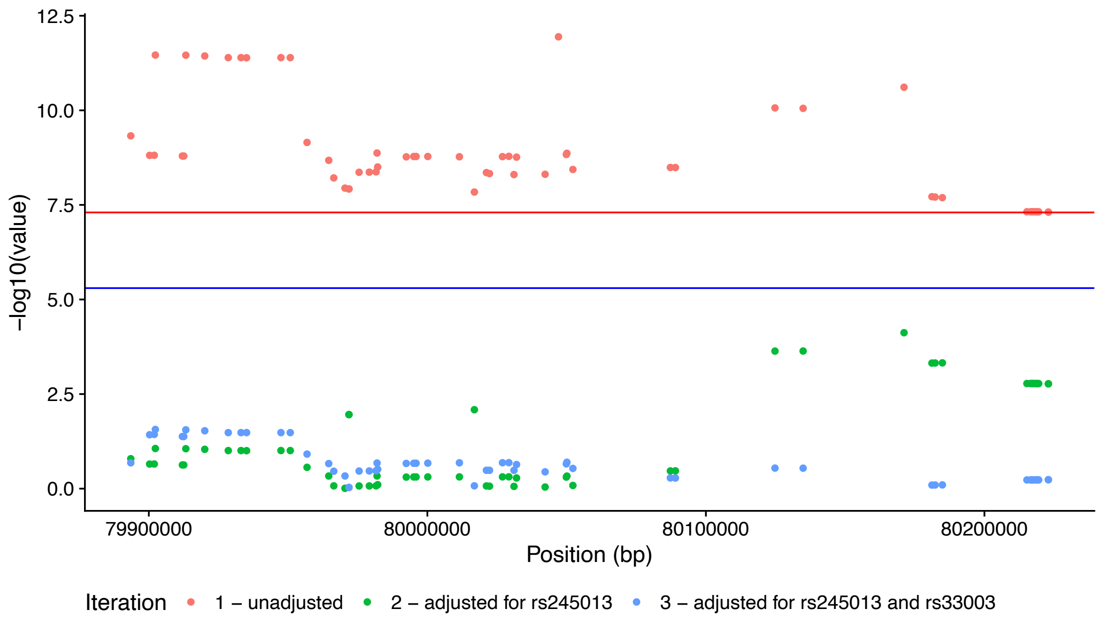

Supplementary Figure 1. Fine mapping of the chromosome 5 candidate region. To determine whether one or several single-nucleotide polymorphism (SNPs) are responsible for the chromosome 5 signal, we iteratively recalculated the regression coefficients of all SNPs per region adjusted for the SNP with the lowest *p*-value in this region. Iteration 1 (red) shows the unadjusted p-values, while the p-values were adjusted for rs374288546 in iteration 2 (green) where six SNPs still have *p*-values below $5x10^{-4}$. Last Iteration 3 (blue) shows *p*-values adjusted for rs374288546 and rs245378 where no other SNP remains significant. Genomic coordinates are given according to GRCh37. Source data are provided as a Source Data file.

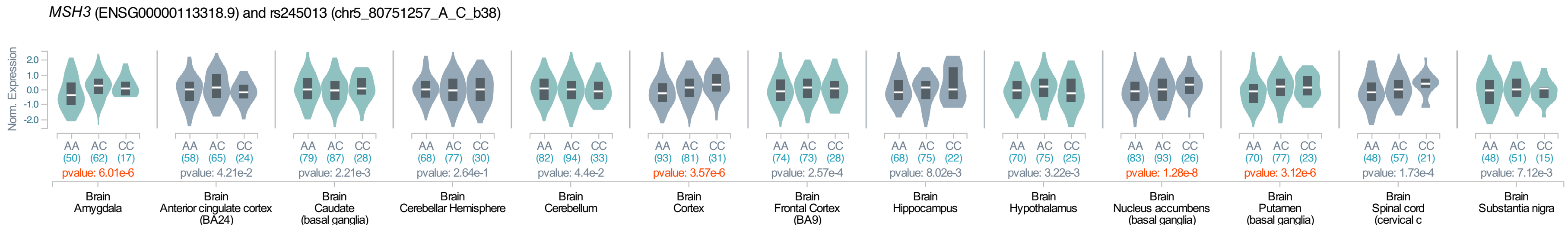

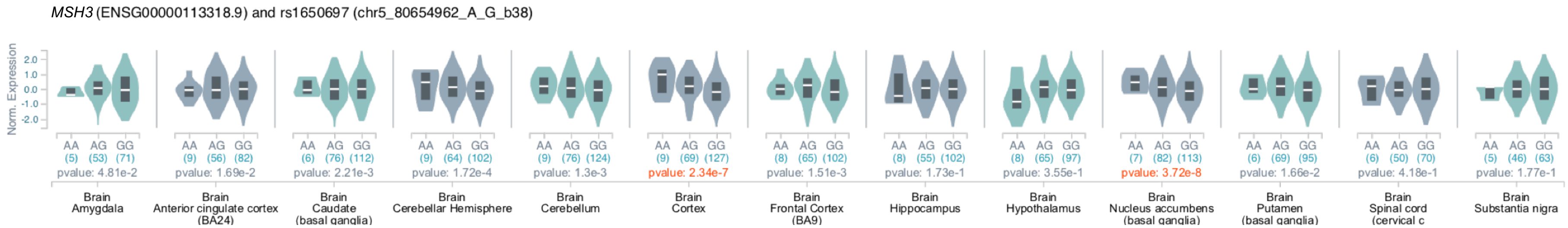

Supplementary Figure 2. Expression quantitative trait locus (eQTL) in rs245013 (upper plots) rs1650697 (lower plots) in brain tissue. The GTEx Portal was queried for tissue-specific association of gene expression with the rs245013and rs1650697 variants revealing significant eQTLs (red). Source data: https://www.gtexportal.org/home/gene/MSH3.

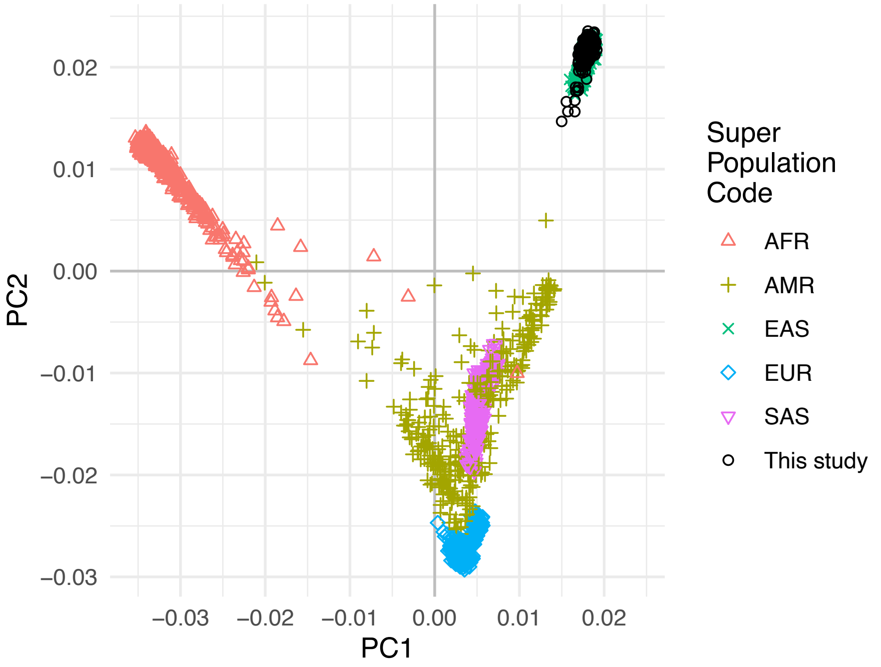

Supplementary Figure 3. Population stratification of our genome-wide association study (GWAS) patient visualized by Principal Component (PC) Analysis plot. All 353 patients clustered together with East Asian (EAS) super population samples in PCA based on shared variants with 1000 Genomes Phase 3 reference haplotypes. AFR: African; AMR: Admixed American; EUR: European; SAS; South Asian. Source data are provided as a Source Data file.

Supplementary References

1. Makino, S. *et al.* Reduced neuron-specific expression of the TAF1 gene is associated with X-linked dystonia-parkinsonism. *Am. J. Hum. Genet.* **80**, 393–406 (2007).
2. Domingo, A. *et al.* New insights into the genetics of X-linked dystonia-parkinsonism (XDP, DYT3). *Eur. J. Hum. Genet.* **23**, 1334–40 (2015).
3. Bragg, D. C. *et al.* Disease onset in X-linked dystonia-parkinsonism correlates with expansion of a hexameric repeat within an SVA retrotransposon in TAF1. *Proc. Natl. Acad. Sci. U. S. A.* **114**, E11020–E11028 (2017).
4. Westenberger, A. *et al.* A hexanucleotide repeat modifies expressivity of X-linked dystonia parkinsonism. *Ann. Neurol.* ana.25488 (2019) doi:10.1002/ana.25488.
5. Karczewski, K. J. *et al.* Variation across 141,456 human exomes and genomes reveals the spectrum of loss-of-function intolerance across human protein-coding genes. *bioRxiv* 531210 (2019) doi:10.1101/531210.
6. Jaffe, A. E. *et al.* Developmental and genetic regulation of the human cortex transcriptome illuminate schizophrenia pathogenesis. *Nat. Neurosci.* **21**, 1117–1125 (2018).
7. Kerimov, N. *et al.* eQTL catalogue: A compendium of uniformly processed human gene expression and splicing QTLs. *bioRxiv* 2020.01.29.924266 (2020) doi:10.1101/2020.01.29.924266.
8. The Genotype-Tissue Expression (GTEx) Project; https://gtexportal.org/; 11/30/20.
