# Supplementary material for: Identifying genetic modifiers of age-associated penetrance in X-linked dystonia-parkinsonism
Source: Nat Commun. 2021 May 28;12:3216. doi: 10.1038/s41467-021-23491-4 (PMC8163740; doi:10.1038/s41467-021-23491-4)
Supplement: Supplementary file 3 — Description of Additional Supplementary Files [file 41467_2021_23491_MOESM3_ESM.pdf]

Description of Additional Supplementary Files

File Name: Supplementary Data 1
Description: . Expression quantitative trait loci (eQTL) query of the lead single-nucleotide polymorphisms (SNPs) from our study using the Genotype-Tissue Expression (GTEx) Portal (https://www.gtexportal.org/home/gene/MSH3 and https://www.gtexportal.org/home/gene/PMS2).[8]

File Name: Supplementary Data 2
Description: Lead single-nucleotide polymorphisms (SNPs) from our study as cis-acting expression quantitative trait loci (eQTL) for MSH3 or PMS2 across 10 brain regions (the cerebellum (CRBL), frontal cortex (FCTX), hippocampus (HIPP), medulla (MEDU), occipital cortex (OCTX), putamen (PUTM), substantia nigra (SNIG), temporal cortex (TCTX), thalamus (THAL), and white matter (WHMT) available from the United Kingdom Brain Expression Consortium (UKBEC) dataset (http://www.braineac.org/).
