# Supplementary material for: Identifying genetic modifiers of age-associated penetrance in X-linked dystonia-parkinsonism
Source: Nat Commun. 2021 May 28;12:3216. doi: 10.1038/s41467-021-23491-4 (PMC8163740; doi:10.1038/s41467-021-23491-4)
Supplement: Supplementary file 6 — Reporting Summary [file 41467_2021_23491_MOESM6_ESM.pdf]

nature research

Corresponding author(s): Ana Westenberger, Inke R. König, Christine Klein

Last updated by author(s): Apr 7, 2021

Reporting Summary

Nature Research wishes to improve the reproducibility of the work that we publish. This form provides structure for consistency and transparency in reporting. For further information on Nature Research policies, see our Editorial Policies and the Editorial Policy Checklist.

Statistics

For all statistical analyses, confirm that the following items are present in the figure legend, table legend, main text, or Methods section.

n/a	Confirmed	
☐	☒	The exact sample size (n) for each experimental group/condition, given as a discrete number and unit of measurement
☐	☒	A statement on whether measurements were taken from distinct samples or whether the same sample was measured repeatedly
☐	☒	The statistical test(s) used AND whether they are one- or two-sided<br>*Only common tests should be described solely by name; describe more complex techniques in the Methods section.*
☒	☐	A description of all covariates tested
☐	☒	A description of any assumptions or corrections, such as tests of normality and adjustment for multiple comparisons
☐	☒	A full description of the statistical parameters including central tendency (e.g. means) or other basic estimates (e.g. regression coefficient) AND variation (e.g. standard deviation) or associated estimates of uncertainty (e.g. confidence intervals)
☐	☒	For null hypothesis testing, the test statistic (e.g. F, t, r) with confidence intervals, effect sizes, degrees of freedom and P value noted<br>*Give P values as exact values whenever suitable.*
☒	☐	For Bayesian analysis, information on the choice of priors and Markov chain Monte Carlo settings
☒	☐	For hierarchical and complex designs, identification of the appropriate level for tests and full reporting of outcomes
☐	☒	Estimates of effect sizes (e.g. Cohen's d, Pearson's r), indicating how they were calculated

Our web collection on statistics for biologists contains articles on many of the points above.

Software and code

Policy information about availability of computer code

Data collection	No software was used.
Data analysis	PLINK1.9; SHAPEIT2; IMPUTE2; SNPTEST2; R 3.6; MAGMA v1.08

For manuscripts utilizing custom algorithms or software that are central to the research but not yet described in published literature, software must be made available to editors and reviewers. We strongly encourage code deposition in a community repository (e.g. GitHub). See the Nature Research guidelines for submitting code & software for further information.

Data

Policy information about availability of data

All manuscripts must include a data availability statement. This statement should provide the following information, where applicable:

- Accession codes, unique identifiers, or web links for publicly available datasets
- A list of figures that have associated raw data
- A description of any restrictions on data availability

The authors declare that all data supporting the findings of this study are included in the article and its supplementary information files or are available from the corresponding authors upon reasonable request. Furthermore, the authors used the following databases: i) The Haplotype Reference Consortium (HRC) reference panel (release 1.1, The European Genome-phenome Archive EGAD00001002729; https://ega-archive.org/datasets/EGAD00001002729), ii) The Genome Aggregation Database (gnomAD) (v2.1.1, https://gnomad.broadinstitute.org/gene/ENSG00000113318?dataset=gnomad_r2_1), iii) The Genotype-Tissue Expression (GTEx) project (https://www.gtexportal.org/home/gene/MSH3#eQTLBlock, Analysis Release V8 (dbGaP Accession phs000424.v8.p2)), iv) the BrainSeq dataset from the eQTL (expression quantitative trait locus) Catalogue (https://www.ebi.ac.uk/eqtl/Studies/), v) as well as the United Kingdom Brain Expression Consortium (UKBEC) (http://www.braineac.org/). The GWAS summary statistics (source data for Figure 1) are available through the GWAS Catalog (ftp://ftp.ebi.ac.uk/pub/databases/gwas/summary_statistics/GCST90014263). For Figures 2, 3b, and 3c, Supplementary Tables 2, 3, 4, and 6, and Supplementary Figures 1 and 3 Source

Data are provided with this paper.

Field-specific reporting

Please select the one below that is the best fit for your research. If you are not sure, read the appropriate sections before making your selection.

- [x] Life sciences
- [ ] Behavioural & social sciences
- [ ] Ecological, evolutionary & environmental sciences

For a reference copy of the document with all sections, see nature.com/documents/nr-reporting-summary-flat.pdf

Life sciences study design

All studies must disclose on these points even when the disclosure is negative.

Sample size	We performed the genome-wide association study in 353 participants. Samples from 37 more recently enrolled XDP patients and 162 healthy Filipino control individuals were investigated in post-GWAS genetic analyses. This sample size was based on the number of X-linked dystonia-parkinsonism patients that we were able to examine and recruit. The sample size was sufficient due to the genetic homogeneity among our patients and the effect of the identified modifiers.
Data exclusions	Initially, DNA samples from 458 men with XDP were used for analyses in this study. Upon genome-wide single-nucleotide polymorphism genotyping, 8 samples with low genotyping quality and 97 patients related to the included individuals were removed before further analyses.
Replication	At this point, we are not in a position to replicate our findings as we still have not collected a large enough cohort of patients unrelated to the patients analyzed in the submitted work. We estimate that we will recruit enough patients for replication in two to five years.
Randomization	Randomization is not applicable for genome-wide association studies given that this is an observational epidemiological study and not a clinical trial.
Blinding	Blinding is not applicable for genome-wide association studies given that this is an observational epidemiological study and not a clinical trial.

Reporting for specific materials, systems and methods

We require information from authors about some types of materials, experimental systems and methods used in many studies. Here, indicate whether each material, system or method listed is relevant to your study. If you are not sure if a list item applies to your research, read the appropriate section before selecting a response.

Materials & experimental systems

n/a	Involved in the study	
x		Antibodies
x		Eukaryotic cell lines
x		Palaeontology and archaeology
x		Animals and other organisms
	x	Human research participants
x		Clinical data
x		Dual use research of concern

Methods

n/a	Involved in the study	
x		ChIP-seq
x		Flow cytometry
x		MRI-based neuroimaging

Human research participants

Policy information about studies involving human research participants

Population characteristics	Our study population consists of patients affected by X-linked dystonia-parkinsonism (XDP). Their mean age at onset (±standard deviation (SD)) was 41.8±8.4 (range: 21-67) years. All of the patients are male, and they all carry the disease-causing variant (the SVA retrotransposon insertion in exon 32 of the TAF1 gene).
Recruitment	Patient recruitment and sample collection were mainly achieved in the Philippines. In multiple fieldwork visits to Panay island, where the majority of the patients live, members of the XDP Study Group from the Philippines and in some cases, members of the Lübeck team personally examined study participants, investigated their family history, and collected blood samples. There was no self-selection bias or other biases that may have influenced the results.
Ethics oversight	Participant enrollment and data analysis were approved by the Ethics Committees of the University of Lübeck, Germany, Massachusetts General Hospital, Boston, USA, Metropolitan Medical Center, Manila, Philippines, and Jose Reyes Medical Center, Manila, Philippines.

Note that full information on the approval of the study protocol must also be provided in the manuscript.
